# The effect of anaerobic digestate as an organic soil fertilizer on the diversity and structure of the indigenous soil microbial and nematode communities

**DOI:** 10.1007/s11356-024-32850-9

**Published:** 2024-03-22

**Authors:** Charitini Nikolaidou, Magkdi Mola, Spiros Papakostas, Vassilis G. Aschonitis, Nikolaos Monokrousos, Panagiotis G. Kougias

**Affiliations:** 1 Soil and Water Resources Institute, Hellenic Agricultural Organization Dimitra, 57001 Thessaloniki, Greece; 2 University Center of International Programmes of Studies, International Hellenic University, 57001 Thessaloniki, Greece; 3 Department of Science and Technology, International Hellenic University, 57001 Thessaloniki, Greece

**Keywords:** Soil quality, Biogas digestate, Ecological networks, Soil nutrients, Microfauna, Organic additive

## Abstract

Anaerobic digestate is a popular soil additive which can promote sustainability and transition toward a circular economy. This study addresses how anaerobic digestate modifies soil health when combined with a common chemical fertilizer. Attention was given to soil microbes and, a neglected but of paramount importance soil taxonomic group, soil nematodes. A mesocosm experiment was set up in order to assess the soil’s microbial and nematode community. The results demonstrated that the microbial diversity was not affected by the different fertilization regimes, although species richness increased after digestate and mixed fertilization. The composition and abundance of nematode community did not respond to any treatment. Mixed fertilization notably increased potassium (K) and boron (B) levels, while nitrate (NO_3_^−^) levels were uniformly elevated across fertilized soils, despite variations in nitrogen input. Network analysis revealed that chemical fertilization led to a densely interconnected network with mainly mutualistic relationships which could cause ecosystem disruption, while digestate application formed a more complex community based on bacterial interactions. However, the combination of both orchestrated a more balanced and less complex community structure, which is more resilient to random disturbances, but on the downside, it is more likely to collapse under targeted perturbations.

**Supplementary Information:**

The online version contains supplementary material available at 10.1007/s11356-024-32850-9.

## Introduction

Biogas plants generate substantial quantities of a by-product with no market value, called “digestate.” In the European Union (EU) alone, approximately 222–258 Mt of digestate was produced in 2021, and this volume is projected to double by 2030, as reported by the European Biogas Association (EBA 2022). The management of digestate clearly poses a significant environmental challenge, since disposal methods like land spreading or landfilling are unsustainable and pose the problem of finding available land outlets (WRAP and Environment Agency 2009). EU has committed itself to pursue long-term environmental goals (Constanze 2020) entailed a reevaluation of how this specific substrate could be efficiently utilized to benefit both agriculture and the environment. In view of this, the use of digestate as a soil improver and/or as fertilizer in agriculture has gained popularity not only within EU member states but also within the scientific community (Koszel and Lorencowicz 2015; Wang et al. 2023). EU has thus issued regulations that encourage the sustainable application of digestate. Notably, the Fertilizing Products Regulation (EU 2019/1009) recognizes digestate as a component of organic fertilizing products in the EU, granting access to the internal market and allowing it to compete on equal terms with mineral fertilizers. The Circular Economy Action Plan (EU 2020/98) emphasizes the urgent need to close the agricultural nutrient loop by increasing digestate use, while discouraging the use of chemical fertilizers.

Several studies have demonstrated the positive impact of digestate application on soil structure and plant growth, promoting its use as an amendment or fertilizer. For example, Tan et al. (2021) presented evidence that digestate application improves soil structure and the composition of soil dissolved organic matter, while at the same time increases the content of the beneficial humic and fulvic-like components. Additionally, digestate’s stability, low impurity levels, and high nitrogen (N) content make it a practical choice for soil restoration purposes (Carabassa et al. 2020). Furthermore, digestate’s slow-release properties help prevent nutrient leaching (Möller and Müller 2012), thus reducing the risk of water contamination and complying to EU regulations regarding water quality and nutrient management. Digestate’s effectiveness as a fertilizer is primarily attributed to its high nutrient content since it is rich in nitrogen (N), phosphorus (P), and potassium (K), making it highly effective for promoting plant growth (Lee et al. 2021). This has the potential to reduce the reliance on expensive chemical fertilizers and mitigate their adverse effects on ecosystems, including eutrophication and heavy metal accumulation (Savci 2012)*.* Additionally, due to its high-water content (92%) (EBA 2015), anaerobic digestate could potentially be used for irrigation, contributing to the reduction of arid zones, which are becoming more prevalent as a result of climate change (Baştabak and Koçar 2020).

Studying the overall biological health of soil is of paramount importance when introducing a new organic product, such as digestate, into complex agricultural systems. Soil microbial communities play a crucial role in maintaining soil fertility and ecosystem functioning. Therefore, a comprehensive assessment of the indigenous soil microbiota provides valuable insights into the potential impacts of organic amendments like digestate (Karimi et al. 2022). Research has shown that digestate application can significantly influence soil microbial communities. For example, a study by Sapp et al. (2015) revealed that digestate application altered soil microbial diversity and community structure, inducing changes in the abundance of specific microbial groups. Another study reported a significant increase in microbial activity and physiological diversity after digestate application in a metal-polluted site (Garcia-Sánchez et al. 2015). Oppositely, Johansen et al. (2013) found that both anaerobically digested materials tested in a microcosm experiment only caused mild and temporary changes in total soil microbial biomass, function, and community structure. Despite all these findings, the majority of the studies focused on assessing the total microbial biomass or the microbial biomass of broader microbial groups (Karimi et al. 2022), without delving into biodiversity metrics nor the dynamic interactions within the communities using more advanced molecular techniques. Novel next generation sequencing techniques will aid in elucidating the dynamic relationships that are altered or created between indigenous and introduced microbial communities in order to optimize the use of digestate as fertilizer/amendment.

Soil nematodes, a taxonomic group which is often overlooked, are the most abundant metazoans on Earth. Nematodes have long been used as bioindicators due to their well-studied taxonomy and feeding habits and because they capture well the disturbance regime of a habitat (Lu et al. 2020). Soil nematodes play crucial roles in soil food webs as consumers at various trophic levels, contributing to decomposition and nutrient cycling. They engage in competitive (Neher 2001) or even mutualistic interactions with bacteria (Kiontke and Fitch 2013) and fungi (Fitter and Garbaye 1994), thereby regulating their populations. Consequently, through this population control, nematodes indirectly influence various processes performed by these microorganisms, including nutrient mineralization, disease prevention, and pollutant degradation (Bongers and Ferris 1999). It is evident that understanding how digestate affects nematode abundance and diversity will provide a more holistic view of its effects on broader soil ecological functions. However, research studies investigating the impact of digestate application on nematode communities and their interactions with other soil biota are limited (Karimi et al. 2022; van Midden et al. 2023).

Organic fertilizers usually favor soil microbial communities and often positive effects on both their abundance and diversity is observed (Bebber and Richards 2022). Chemical fertilizers, by contrast, often reduce microbial growth and biodiversity (Sabir et al. 2021). Combining organic and chemical fertilizers can improve plant yield, as well as bacterial and nematode biomass and diversity (Su et al. 2021). To our knowledge, no studies have explored the combined effects of digestate and chemical fertilizers. We propose that using chemical ammonium fertilizers in conjunction with digestate will enrich the digestate with nitrogen, creating a complete fertilizer that includes all necessary macroelements and trace elements.

The central premise of this investigation posits that the application of digestate will augment the diversity within microbial and nematode communities. Furthermore, it is postulated that the synergistic use of digestate alongside chemical fertilizers will engender alterations in the composition and dynamics of these communities. The research objectives are thus delineated to evaluate the impact of digestate and chemical fertilization on (1) the eubacterial community, initially at the phylum level, subsequently delving into a detailed analysis at the genus level; (2) the nematode community; (3) the soil’s chemical attributes; and (4) the stability of community networks, as delineated by the interrelations between nematodes and Eubacteria. A mesocosm pot experiment, utilizing soil from a region of intensive cultivation, was employed to meet these objectives. This single-system experimental design was chosen to exclusively focus on the direct impacts of the soil amendment. This approach contrasts with a plant-soil system experiment, where the influence of plant root exudates on the soil’s microbial and nematode communities may obscure the direct effects of the soil amendment.

## Materials and methods

### Experimental design

Soil was collected from an intensively cultivated area located in ELGO-Dimitra’s campus in Thermi, Thessaloniki. Τhe soil was passed through a 2-mm sieve to remove any roots and twigs and was distributed into 2-L pots. At the same time the whole fraction of anaerobic digestate (before mechanical separation) was collected from a local full-scale biogas plant (Biogas Lagada S.A) operating in mesophilic conditions. The feed substrate of the digester consisted of agricultural and livestock waste, while the collected digestate was previously pasteurized at 70 °C for 1–2 h to inactivate pathogens. The physicochemical characteristics of both soil and digestate are shown in Table 1. Ammonia nitrate (40.5–0-0) was chosen as conventional fertilizer. Α fertilizer recommendation of 20–25 nitrogen (N) units was generated based on the soil nitrogen demand and the restrictions permitted by European and National legislation. However, applying a digestate dosage containing 20–25 units of nitrogen to each pot would be an unrealistic scenario due to the low total Kjeldahl nitrogen (TKN) of digestate, since the amount of digestate necessary to meet the units would be too high. For this reason, digestate was applied in a realistic dose of 5 N units per pot for all treatments that included digestate. In total four different fertilization treatments with five replicates were examined and compared: treatment with conventional fertilizer (20 N units), treatment with digestate (5 N units), treatment with a mixture of digestate and conventional fertilizer (20 N units of conventional fertilizer + 5 N units of digestate), and treatment without fertilization, control (0 N units).

**Table 1:** The physicochemical properties of soil and digestate

Physicochemical properties	Soil	Digestate
Sand:clay:silt (%)	40:24:36	-
pH	7.27	8.2
Ec (mS/cm)	0.410	20.7
Organic matter (%)	1.83	4.71
CaCO3 (%)	4.8	-
TKN (%)	-	0.46
NO_3_^−^ N (%)	13.9	-
NH_4_^+^–N (%)	-	0.36
TON (%)	-	0.1
P (ppm)	7.1	514
K (ppm)	266	3852
Mg (ppm)	278	437
Fe (ppm)	1.5	112
Zn (ppm)	0.5	20.5
Mn (ppm)	3.14	22
Cu (ppm)	1.2	5.3
B (ppm)	0.5	4.7

Both fertilizing components were manually distributed to the pots and quickly integrated into the soil by mixing to achieve homogeneous dispersion and prevent nitrogen volatilization. Following application, all pots were irrigated twice a week to ensure that the soil’s water content was constantly equal to the soil’s water holding capacity. Water holding capacity was determined by saturating 100 g of dry soil and measuring the volume of washed off water. A stabilization period of 1 month was considered sufficient for soil organisms to assimilate nutrients and to allow changes of microbial and nematode community composition to occur. The pots were kept at a temperature range of 21–24 °C under natural light. After 1 month, soil samples were collected (0–20 cm depth) from each pot for molecular and nematode analyses.

### Physicochemical analyses

Soil samples were sieved with a 2-mm sieve to break down soil aggregates and air dried for 48 h at 25 °C. The mechanical composition of soil was measured according to the Bouyoucos method (1962). A conductivimeter was used to measure EC (APHA 2017), and a pH-meter was used for pH determination (soil’s pH was measured in the saturated soil paste). Organic matter was measured by the wet oxidation method using potassium dichromate (Walkley and Black 1934) and calcium carbonate (CaCO_3_) by the volumetric calcimeter method (Allison and Moodie 2016). Total Kjeldahl nitrogen (TKN) and ammonium (NH_4_^+^)–N were measured by the Kjeldahl method (APHA 2017) and nitrate (NO_3_^−^)–N by using KCl 0.5 mol/L (APHA 2017), while P was measured according to the Olsen method with ascorbic acid (Watanabe and Olsen 1965). The concentrations of K, Mg, Fe, Zn, Mn, and Cu were determined by ICP (Perkin Elmer Optical Emission Spectrometer, OPTIMA 2100 DV, Waltham, MA, USA) (Chyla and Zyrnicki 2000). Boron (B) was determined according to the method of azomethine-H (Wolf 1974).

### Extraction of soil nematodes and identification

Soil nematodes were extracted from 150 mL of soil following Cobb’s sieving and decanting method as modified by S’Jacob and van Bezooijen (S’Jacob and Van Bezooijen 1984). The total abundance of nematodes was measured using a Euromex StereoBlue stereoscope (Euromex, Arnhem, the Netherlands). Following quantification, nematodes were preserved in 4% formaldehyde solution, and 100 nematodes were randomly picked from each sample and identified through microscopy down to genus level using Bongers’ identification key (Bongers 1994).

### DNA extraction and library preparation

DNA extraction was performed using 250 mg of soil with E.Z.N.A.® Soil DNA Kit (Omega Bio-tek, Norcross, GA, USA) following manufacturer’s instructions. Quantification and quality check of the extracts were performed using a Quawell Q3000 microvolume spectrophotometer (Quawell Technology, San Jose, USA). Library preparation was performed using the 16S Barcoding Kit 1–24 (Oxford Nanopore Technologies, Oxford, UK) following manufacturer’s instructions. The full-length 16S rRNA bacterial gene was amplified through PCR using the kit’s barcoded primers (27F: 5′-AGAGTTTGATCMTGGCTCAG-3′ and 1492R: 5′-CGGTTACCTTGTTACGACTT-3′) allowing multiplexing. Amplification was conducted using repliQa HiFi ToughMix (Quantabio, Beverly, MA, USA) in Eppendorf Mastercycler Gradient (Eppendorf, Hamburg, Germany) at the following conditions: initial denaturation at 95 °C for 1 min followed by 25 cycles of denaturation (95 °C, 20 s), annealing (55 °C, 30 s), and extension (65 °C, 2 min), with a final extension at 65 °C for 5 min. After amplification followed the PCR product purification with Agencourt AMPure XP beads (Beckman Coulter, CA, USA). The concentration of purified DNA amplicons was determined using the microvolume spectrophotometer.

### Amplicon high-throughput sequencing and data processing

All libraries (100 ng of DNA in 10 µL buffer) were mixed with 1 µL of rapid adapter, 34 µL of sequencing buffer, 25.5 µL of loading beads, and 4.5 µL of water. The sequencing solution was then loaded on MinION R9.4.1 SpotON Flow Cell which was controlled through the MinKNOW software v.3.6.5.

Nanopore signals were base called, and sequences were demultiplexed and trimmed for barcode and adapter sequences using Guppy v.3.2.10 (Oxford Nanopore Technologies). On average, 243,479 reads were produced per sample. This sequencing data underwent further processing with Porechop v.0.2.4 (Wick RR, https://github.com/rrwick/Porechop) to remove any remaining adapters. Reads containing internal adapters, in particular, were considered chimeric and were subsequently discarded using the “discard_middle” option. Following this, reads were filtered using the chopper v.0.2.0 tool from the NanoPack2 toolkit (De Coster and Rademakers 2023) in the following manner: (a) We trimmed 25 nucleotides from the start of each sequence (–headcrop 25). This step aimed to enhance the overall quality of the reads, as, using FastQC v.0.12.1 (Andrews S. 2010), we observed relatively lower base calling quality in the initial 20 nucleotides. (b) We allowed for sequences with a length ranging between 1000 and 1600 nucleotides (–minlength 1000 –maxlength 1600). This adjustment further improved the overall quality of the retained reads, following inspection of the mean Q score in relation to read length using MinIONQC v.1.4.2 (Lanfear et al. 2019). (c) In the end, reads with an overall mean quality of 7 or higher were retained (–quality 7).

The filtered reads were classified to taxonomic units using the k-mer matching algorithm of Kraken2 v.2.1.2 (Wood et al. 2019). The SILVA SSU database 138 (https://www.arb-silva.de/documentation/release-138/) of the SILVA ribosomal RNA gene database project (Quast et al. 2012) was used as a reference database for taxonomic classification with a confidence threshold of 90% for taxonomic assignments. Subsequently, Bracken v.2.8 was employed (Lu et al. 2017), a Bayesian method to compute abundances from Kraken-derived taxonomic assignments. The OTU read counts derived from Bracken were normalized for each sample using the median sequencing depth. In the present study we examined the most abundant operational taxonomic units (OTUs) for each sample, using a cut-off value of 0.01% out of the total number of sequences. Raw reads were uploaded to the NCBI’s sequence read archive database (SRA) under the BioProject PRJNA1049417.

### Statistical analysis and network analysis

Microbial community composition was visualized and analyzed using R packages Phyloseq (McMurdie and Holmes 2013). The Shannon index was used to assess α-diversity, while β-diversity was assessed using nonmetric multidimensional scaling (NMDS) analysis using Bray–Curtis dissimilarity. Welch’s *t*-test was performed using STAMP software (Parks and Beiko 2010) to identify significantly differentiated taxa between treatments. *Z*-scores of means and standard deviations of taxa that showed significant differences were calculated per taxon and presented in heatmaps using the pheatmap package (Kolde 2012) in R.

In network analysis, nodes stand for microbial and nematode genera while edges stand for Spearman’s correlation coefficients (> 0.6) between these genera. The correlation matrices were analyzed and visualized with the igraph package (Csárdi et al. 2023). For each network some topological characteristics that describe the interrelationships of bacteria and nematodes were estimated: average path length (APL), graph density, network diameter, average clustering coefficient (avgCC), average connectivity (avgK), and modularity (M). Furthermore, the small-world index (SW) was calculated by comparing estimates of the average path length (APL) and average clustering coefficient (avgCC) calculated from the experimental data and from a set of 1000 simulated random networks with the same size and degree distribution. If SW > 1 the real network was considered small-world (Humphries and Gurney 2008).

To identify potential differences between treatments for the physicochemical properties, a one-way analysis of variance (ANOVA) was performed individually for each variable tested. Post-hoc Tukey tests were carried out for variables that exhibited statistically significant differences to determine which pairs of treatments are significantly different from each other.

To further investigate the relationships between samples, physicochemical properties and bacterial and nematode abundance, a redundancy analysis (RDA) was employed using the vegan package (Oksanen et al. 2017) in R.

## Results and discussion

### Microbial community composition

In total 63 different taxa remained after filtering for further analysis and were identified down to genus level. The average sequencing depth for all samples after normalization was 155,932 read counts.

#### Phylum and genus level insights: multifaceted richness and diversity responses to fertilization regimes

**Fig. 1:**
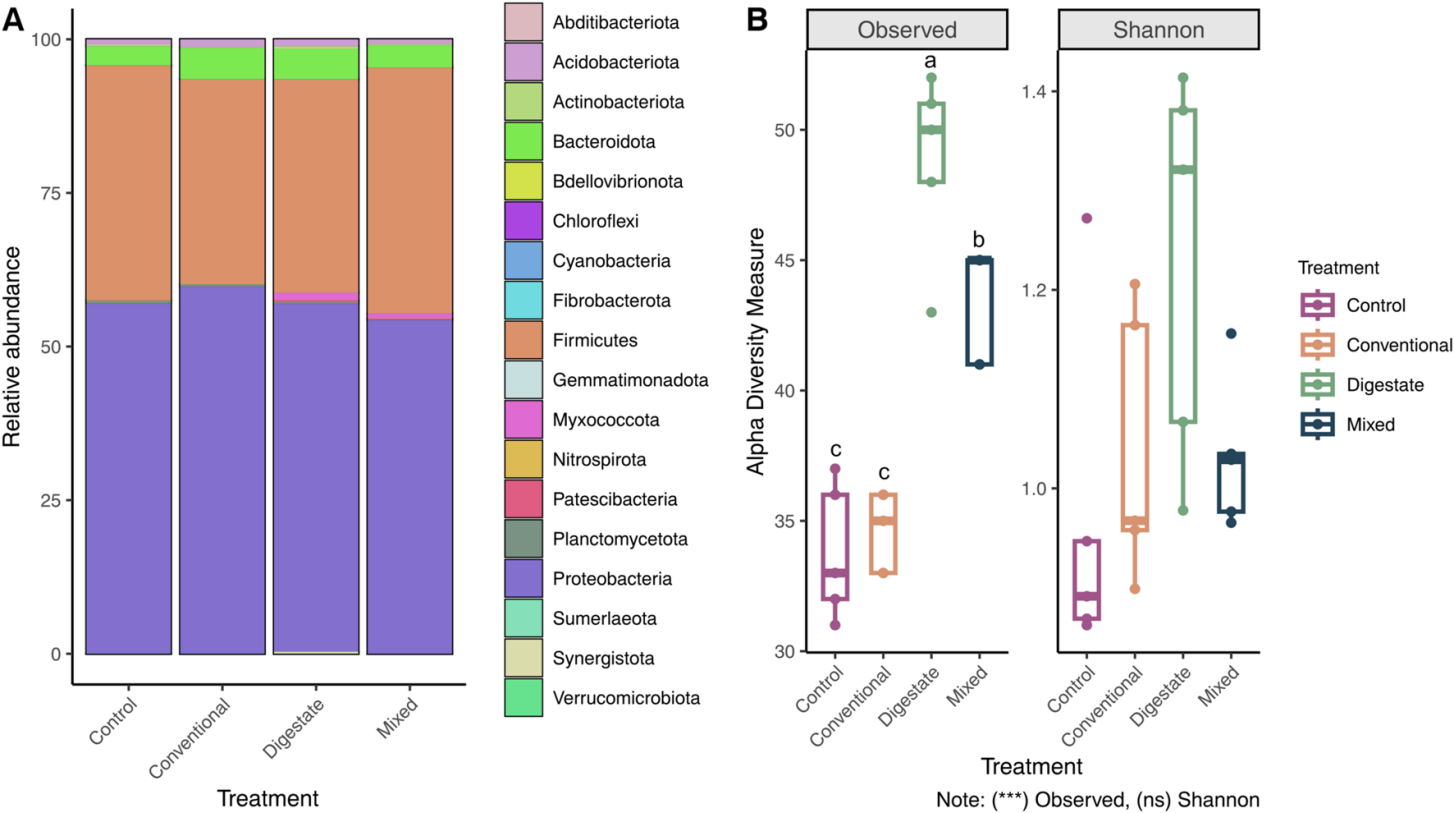
**A** Stacked bar plot representing community composition of different treatments at phylum level. Each bar represents the average relative read counts for each phylum over five replicate libraries for each treatment. **B** Box plots of alpha diversity value distribution for richness and Shannon diversity indices of different treatments are shown. The line inside each box represents the median value and outliers are shown as dots. The top and bottom borders of the box represent the upper and lower quartiles, respectively. The top and bottom extensions of the box indicate the maximum and minimum values within the data. Different letters above boxplots mean significant difference at *p* < 0.001 (***) according to post-hoc Tukey HSD test

Chemical fertilizer, digestate, and their combination did not appear to induce substantial alterations in the soil microbial structure at the phylum level, as the community structure of the control exhibited similarity to that of the other treatment groups (Fig. 1A). The predominant phyla across all communities were Proteobacteria and Firmicutes, while Bacteroidota exhibited a comparatively lower presence. The main representatives of the abovementioned phyla are the genera *Pseudomonas*, *Bacillus*, and *Sphingobacterium*, respectively (Supplementary Table, Fig. S1). *Bacillus* and *Pseudomonas* spp. are ubiquitous plant growth–promoting bacteria in soil ecosystems and they are involved in diverse soil functions including nutrient cycling, enhancing plant growth through phytohormone production, and protecting plants from pathogens and environmental stressors (Saxena et al. 2020; Sah et al. 2021). They also include several denitrifying bacteria. Despite the soil’s initial low nitrate levels (Table 1), their significant presence in all treatments (including the control) could be attributed to the historically high nitrate levels of the intensively cultivated soil. Sphingobacteriales have been positively associated with N_2_O fluxes and high nitrogen availability (Hester et al. 2018). Thus, their higher relative abundance in soils treated with digestate and conventional fertilizer could be attributed to the higher nitrogen inputs. Interestingly, the mixed treatment, despite its higher nitrogen levels, Sphingobacteriales abundance was not promoted, revealing some intricate interactions between the two fertilizers that impact the microbial community. The rest of all other phyla presented very low percentages of relative abundance. Despite the fact that the communities did not differ substantially at the phylum level, the richness at genus level appears to vary between treatments. Additionally, the mixed fertilization treatment presented a lower number of taxa than digestate, indicating that the chemical fertilizer fraction inhibited the establishment of microorganisms introduced by the digestate fraction. Digestate treatment, in particular, presented significantly higher number of genera compared to all other treatments (Fig. 1B), while treatment with chemical fertilizer did not differ from the control, coinciding with other studies (Dincă et al. 2022). The incorporation of digestate increased the relative abundance of Myxococcota phylum, which was represented by the single genus *Anaeromyxobacter* sp. and it was present in both digestate and mixed treatments, although in a smaller proportion in the later. *Anaeromyxobacter* has been lately characterized as diazotroph which can survive under nitrogen deficient environments (Masuda et al. 2020). Its lower dominance in the mixed treatment could be attributed to the sufficiency of nitrogen in mixed fertilized soils. Other newly introduced taxa from digestate belonged to Bacteroidota phylum (Supplementary Table, Fig. S1), which play key role in several biogeochemical functions such as soil fertility, carbon storage, and the degradation of complex carbohydrate-based biomass (Larsbrink and McKee 2020). For example, genus *Crocinitomix* of the phylum Bacteroidota is known to produce catalase, an indispensable enzyme that protects cells from oxidative stress, enhancing soil health (Bowman et al. 2003). In addition, digestate introduced some rare phyla composed of single genera, such as *Chloroflexi*, *Cyanobacteria*, *Gemmatimonadota*, *Synergistota*, *Myxococcota*, and *Patescibacteria* (Supplementary Table, Fig. S1), enhancing the richness and thus the soil functionality and dynamics. Surprisingly, in the mixed treatment the phyla Chloroflexi, Cyanobacteria, and Synergistota were not present at all, suggesting that these particular phyla are suppressed by the chemical fertilizer.

Nonetheless, the Shannon diversity index (Fig. 1B) revealed no significant changes among the different fertilization regimes. This finding implies that, while richness may vary, overall evenness within microbial communities remains consistent when digestate is applied. This stability might suggest that the digestate-introduced species are integrating into the existing microbial ecosystem without dramatically affecting the overall equilibrium of the microbial community structure. This finding shows that the indigenous soil microorganisms are resistant to external disturbances, at least at the digestate dose applied in this study. This lack of increase in diversity has been previously observed by Coelho et al. (2020), who reported that introduced microbial communities from digestate failed to establish when applied in the soil. At this point, it is worth emphasizing that the current study is a short-term experiment that may reflect a transitional state which could shift over the course of time. Meanwhile, longer-term applications of chemical fertilizers have been proven to trigger perpetual changes in microbial diversity. For example, a previous study by Francioli et al. (2016), which focused on the long-term effects, documented a decrease in biodiversity following repeated chemical fertilizer applications. Thus, the widespread use of chemical fertilizers does not exclude a decline of diversity. Further research with longer-term studies is necessary in order to determine whether this transient state will persist or change over time.

#### Genus level NMDS: distinct and overlapping community responses to fertilization

**Fig. 2:**
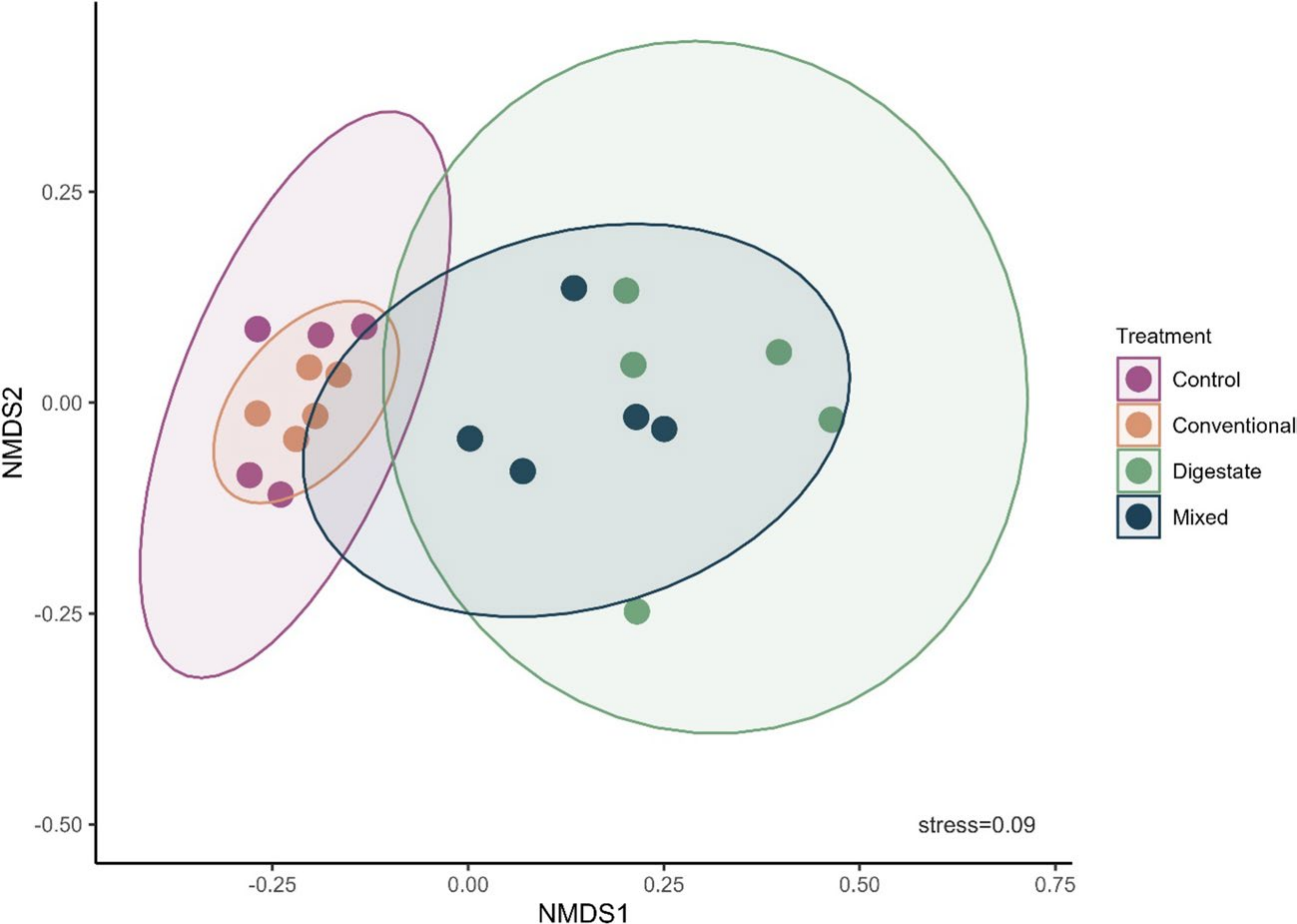
Nonmetric multidimensional scaling (NMDS) of a Bray–Curtis distance matrix describing the distribution of samples based on differences on microbial composition at the genus level. Ellipses represent where 95% of the data are located. Different colors represent different fertilization treatments. More overlap indicates higher degree of similarity between treatments

The nonmetric multidimensional scaling (NMDS) analysis based on the microbial genera reveals intriguing patterns between samples of the different fertilization treatments applied (Fig. 2). Notably, the control and conventional treatments have a significant overlap, indicating that their community compositions are very similar. Oppositely, on the positive side of the *x*-axis, digestate and mixed treatments substantially intersect, with mixed treatment sustaining only a small shared area with control and conventional. Furthermore, the digestate treatment demonstrates the broadest ellipse, suggesting higher variability among samples, whereas the conventional treatment exhibits small ellipse and tightly clustered samples, showing a definite and distinctive microbial community structure. Conventional fertilizer appears to have a considerable influence on the microbial assemblage, resulting in a community with a specific and consistent ecological profile that is entirely affected by nitrogen input. The digestate treatment, on the other hand, implies a more stochastic and variable ecological structuring. Nutrient and microbial inputs originating from digestate reform the microbial community in a less predictable way, encompassing a broader variety of ecological niches. As a result, the microbial population formed after digestate application appears to have a dynamic and diverse ecological profile that is shaped by a combination of factors including both its microbial and nutritional loads. When digestate and chemical fertilizer are combined, this randomness is restrained, and the community is shaped in a more distinct way. This is probably due to the promotion of specific nitrogen-associated taxa that are favored over others when chemical fertilizer is applied.

#### Genus level impacts of fertilization on microbial taxa relative abundance and variability

**Fig. 3:**
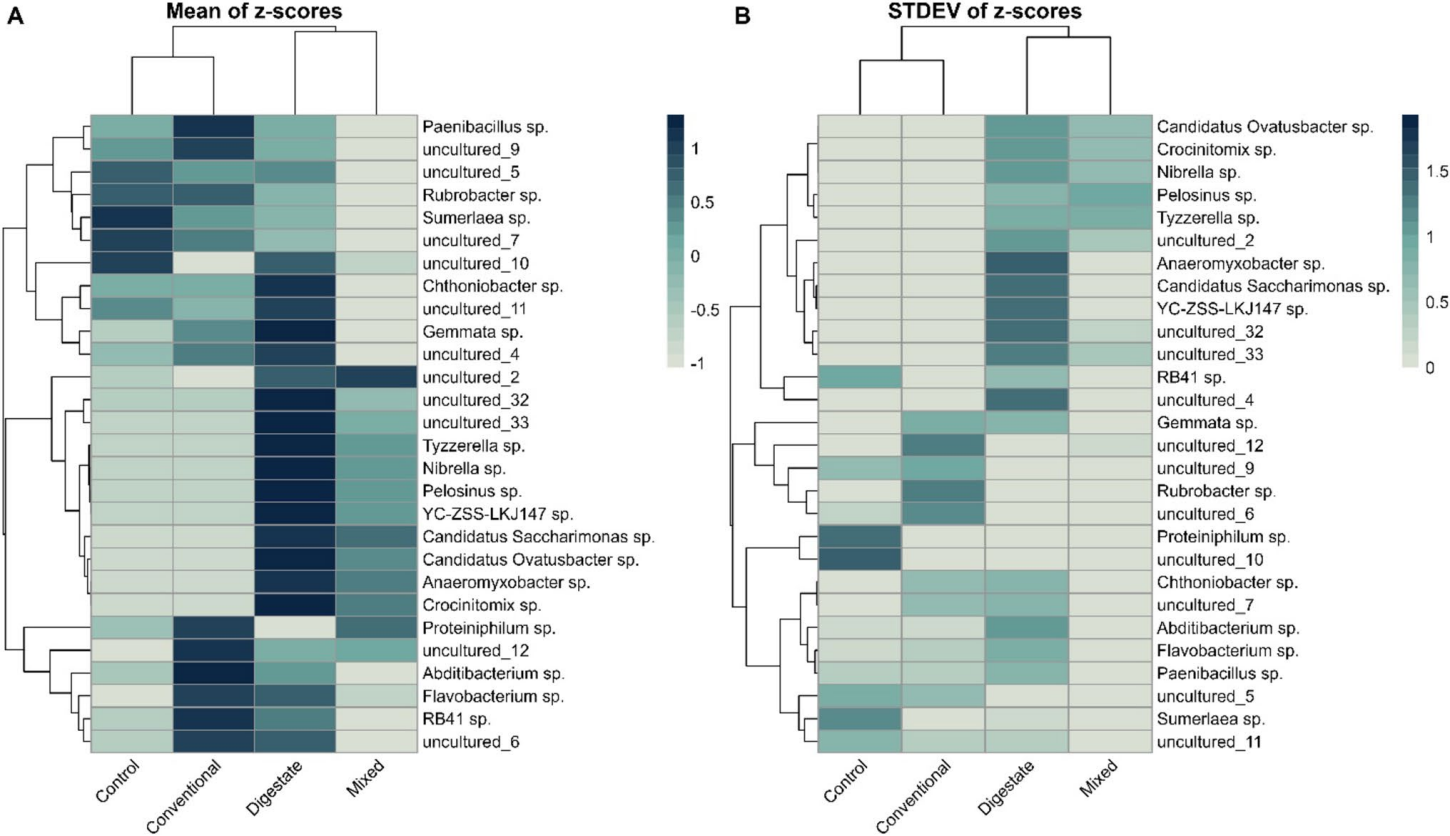
Clustered heatmaps showing **A** the average relative abundances of significantly differentiated microbial taxa in different fertilization treatments as emerged from the pairwise **t**-test analysis and **B** their standard deviations values. Analysis was performed using row-wise *z*-transformed relative abundances

Heatmap analysis of the mean and standard deviation of microbial taxa’s relative abundance revealed a clear separation of treatments in two groups (Fig. 3), aligning with the NMDS pattern. Samples after digestate application displayed higher abundance levels in the majority of significantly differentiated microbial taxa, signifying a greater impact and thus higher stress levels on the soil microbial community in comparison to the other treatments.

More specifically, digestate presented higher relative abundances of Gemmatimonadota (YC-ZSS-LKJ147 sp.) and *Chthoniobacter*, species of which are involved in the transformation of organic carbon compounds in the soil (Sangwan et al. 2004; Mujakić et al. 2022). Higher presence of *Gemmata* sp. belonging to Planctomycetota was also observed in digestate, a phylum that plays considerable role in global carbon and nitrogen cycles (Wiegand et al. 2020). Genus *Pelosinus* is known for its iron-reducing abilities (Hansel et al. 2008), while Candidatus *saccharimonas* rely on amino acids and energy substrates to thrive (Ogunade et al. 2019). All the above-mentioned taxa showed lower presence in control and conventional treatment compared to digestate, possibly due to the lower organic matter and trace element inputs of these soils.

Treatment with conventional fertilizer displayed similar patterns regarding the presence and abundance of microbial taxa with control. Although, significantly higher abundance was presented in the cluster consisting of genera of the Bacteroidota phylum (*Proteiniphilum* sp. and *Flavobacterium* sp.) of the Proteobacteria phylum (uncultured spp.), as well as genera *Abdibacterium* and RB41. Conventional treatment showed comparatively lower abundances than control in several uncultured taxa belonging to *Rhodospirillaceae* family, all members of which have the capacity to fix molecular nitrogen (N_2_) and convert it to ammonium (NH_4_^+^) (Madigan et al. 1984). Hence, a drop in their relative abundance in ammonia nitrate–enriched soils was justified. *Sumerlaea* sp., belonging to the Sumerlaeota phylum, thrived in the control samples with no nitrogen inputs, as they are known to adapt in nitrogen-limited environments (Zhang et al. 2023). However, their abundance decreased in all fertilized soils, likely due to the increased nitrogen availability resulting from fertilization.

On the other hand, the mixed treatment exhibited lower abundances than digestate and conventional fertilizers in almost all significantly differentiated taxonomic groups. This suggests that in the mixed treatment, the combined input of different nitrogen sources and nutrients moderates the higher stress levels induced by the digestate fraction, while simultaneously introducing new microbial taxa not present in the conventional treatment. This contributes to the increase of community richness within the mixed treatment while avoiding high disturbance levels.

Corresponding standard deviations of the significantly differentiated taxa are depicted in the heatmap of Fig. 3B. It is evident that digestate treatment presented high standard deviation in the majority of taxa compared to the other treatments. This increased variability suggests that when digestate is applied to soil, it has a more diverse and less consistent impact on the microbial community. Notably, in a comprehensive review on the worldwide impact of digestates on soil microbial quality, it was found that in 7% of the studies comparing the effects of digestates and chemical fertilization, no definitive conclusions were reached due to the variability in the results obtained (Karimi et al. 2022). Phyla with higher abundance levels in digestate, such as Myxococcota, Gemmatimonadota, and Patescibacteria as well as members of Proteobacteria and Bacteroidota, responded in a more diverse manner to the digestate additive, indicating higher biological heterogeneity within these groups. However, when digestate and chemical fertilizer are applied in combination, it appears to reduce the biological heterogeneity within these groups, leading to a more specific community structure. The increased unpredictability of certain taxa’s responses upon digestate application is one of the primary challenges that must be addressed during the implementation of soil improvement practices.

### Nematode community

**Fig. 4:**
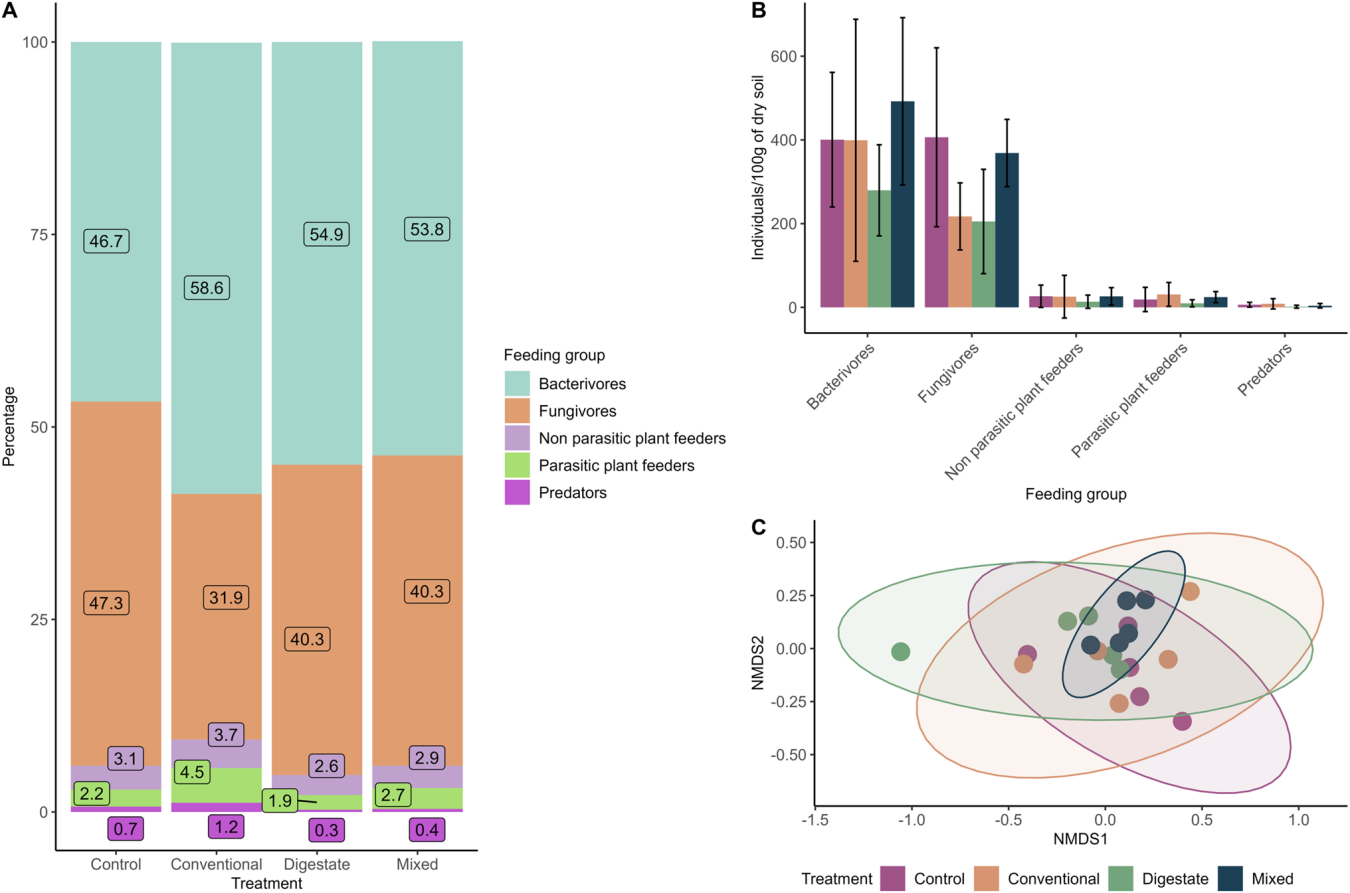
**A** Bar plots showing the percentage abundance of all nematode feeding groups detected in different fertilization treatments. The numbers indicate the percentage of each feeding group in the corresponding treatment. Different colors are assigned to each feeding group. **B** Mean values of the nematode trophic group abundances (individuals/100 g of dry soil) in each fertilization treatment. Error bars show the standard deviation. **C** Nonmetric multidimensional scaling (NMDS) of a Bray–Curtis distance matrix describing the distribution of samples based on differences on nematode composition and abundance. Ellipses represent where 95% of the data are located. More overlap indicates higher degree of similarity between treatments. Different colors represent different fertilization treatments for B and C

Nematode identification through microscopy revealed 16 different genera in the control and conventional treatments, 14 in the digestate treatment, and 15 in the mixed. All genera were designated to five feeding groups (bacterivores, fungivores, predators, non-parasitic, and parasitic plant feeders) based on their feeding habits (Fig. 4A, 4B). The NMDS for the community composition and abundance of nematodes revealed no distinct patterns as samples from all treatments showed substantial scattering (Fig. 4C). All treatments presented wide ellipses except the mixed, samples of which are tightly depicted in a narrower ellipse. This indicates that while conventional fertilizer and digestate lead to random community structures, their combination results in a more defined pattern. One-way ANOVA did not show any significant differences between treatments in none of the five feeding groups, possibly due to the high variability observed within the data, as evidenced by the high standard deviations (Fig. 4B). This result could be attributed to the short timeframe of this experiment, since many free-living soil nematodes have longer lifespans than bacteria ranging from few days to years (Gems 2000). Further experiments need to be conducted over extended periods of time in order to understand the long-term effects of fertilization on nematode populations.

The nematode community composition after the application of different fertilizers is depicted in Fig. 4A. All fertilized soils present higher fungivores/bacterivores ratios compared to the unfertilized control that has almost equal percentages. Productive agricultural soils often exhibit fungal to bacterial biomass ratios close to 1:1 (Ingham et al. 2000). The increasing number of bacterivores in fertilized treatments is related to a possible increase of bacterial populations. Given the addition of nitrogen via fertilization, this bacterial development is justified**,** since bacteria have higher nitrogen demands than carbon demanding fungi**.** As a result, the added nitrogen may have promoted bacterial development, affecting the bacterivores/fungivores ratio in the fertilized soils. The conventional treatment displayed a higher percentage of parasitic plant feeders than the other treatments, although this difference was not statistically significant. However, a distinct trend is presented indicating that parasitic plant feeders are suppressed in all soils treated with digestate, either alone or in combination with chemical fertilizers. The nematicidal activity of biogas digestate against plant parasitic nematodes has been reported before and it may be associated with bioactive fermentative compounds/metabolites (Karimi et al. 2022; Oldani et al. 2023).

### Effects of soil chemical properties

**Fig. 5:**
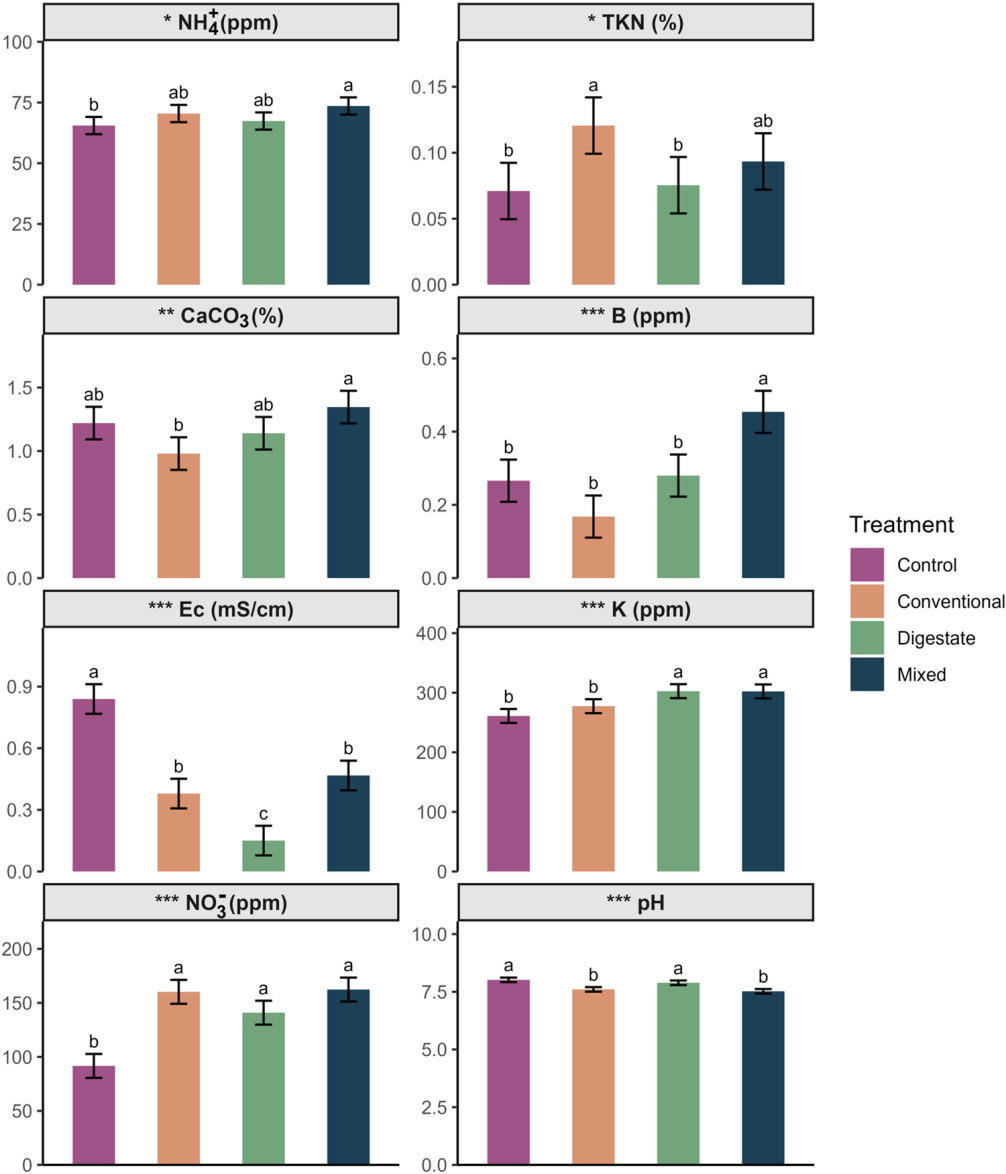
Bar plots of mean values (± standard deviation) of significantly differentiated soil chemical properties after application with different fertilizers as emerged form one-way ANOVA. Different letters above bars denote significant differences among treatments based on Tukey post hoc test (*: *p* < 0.05; **: *p* < 0.01; ***: *p* < 0.001, for all cases *n* = 5). NH4 + , ammonium; TKN, total Kjeldahl nitrogen; CaCO3 − , calcium carbonate; B, boron; Ec, electrical conductivity; K, potassium, NO3 − , nitrate; pH. Corresponding units are shown in parentheses next to the title. Different colors represent different fertilization treatments

Beyond biological traits, soil’s chemical properties are also critical in determining its quality and functionality. The significantly differentiated chemical properties in the various fertilization regimes are shown in Fig. 5. More specifically, significantly higher levels of available K were presented in soils treated with digestate and mixed. Higher potassium availability can support microbial metabolic functions, increase enzyme activity, and trigger responses to osmotic stress and nutrient limitation improving soil homeostasis (Stautz et al. 2021). Boron, an essential micronutrient, presented higher concentrations in the mixed treatment, while all other treatments showed significantly lower levels. Notably, a previous study reported that higher boron levels are positively correlated with increased enzyme activities of soil urease, phosphatase, and dehydrogenase (Bilen et al. 2011). The observed increase in boron levels following the combined application of the chemical fertilizer and digestate could be attributed to a potential interaction between digestate and the ions produced by the hydrolysis of ammonium nitrate. More specifically, ions from ammonium nitrate substitute boron, as they bind to the organic compounds of the digestate, permitting the release of boron ions into the soil solution. TKN was higher in conventional treatment due to the addition of ammonium nitrate to the soil. This increase is likely due to higher organic nitrogen rather ammonium, as ammonium levels were similar to the control. The readily available chemical fertilizer possibly stimulated microbial activity, accelerating soil organic matter decomposition and consequent release of organic nitrogen compounds in the soil. Ec was also a main factor influencing community composition, presenting significantly higher values in control that reflect high levels of salts and ions in the soil solution. In contrary, all fertilized soil exhibited lower Ec, with the soils treated with digestate presenting the lowest values.

Other chemical properties included NH_4_^+^, CaCO_3_^−^, NO_3_^−^, and pH. However, these factors were not the primary drivers of soil community composition, as will be discussed below. Interestingly, both NH_4_^+^ and NO_3_^−^ exhibited higher values in all fertilization treatments, regardless of the variations in the initial nitrogen units applied. This suggests that the addition of 5 N units from digestate was equivalent in terms of nitrogen availability to the 20 N units from chemical fertilizer and the 25 N units from the mixed fertilizer treatment. Although ammonium nitrate fertilizer has a higher initial nitrogen load than digestate, a portion of the nitrates may be immobilized by soil microorganisms, reducing their availability in the soil solution. Oppositely, digestate contains organic nitrogen, which is not immediately available to microbes and is not directly assimilated. Instead, it undergoes a slower conversion process into nitrate through nitrification by soil microorganisms. Combined application of chemical fertilizer and digestate increases microbial biomass in the soil, which, along with the carbon input from digestate, accelerates nitrate immobilization rate (Li et al. 2020), preventing nitrate concentration of soil to increase further.

**Fig. 6:**
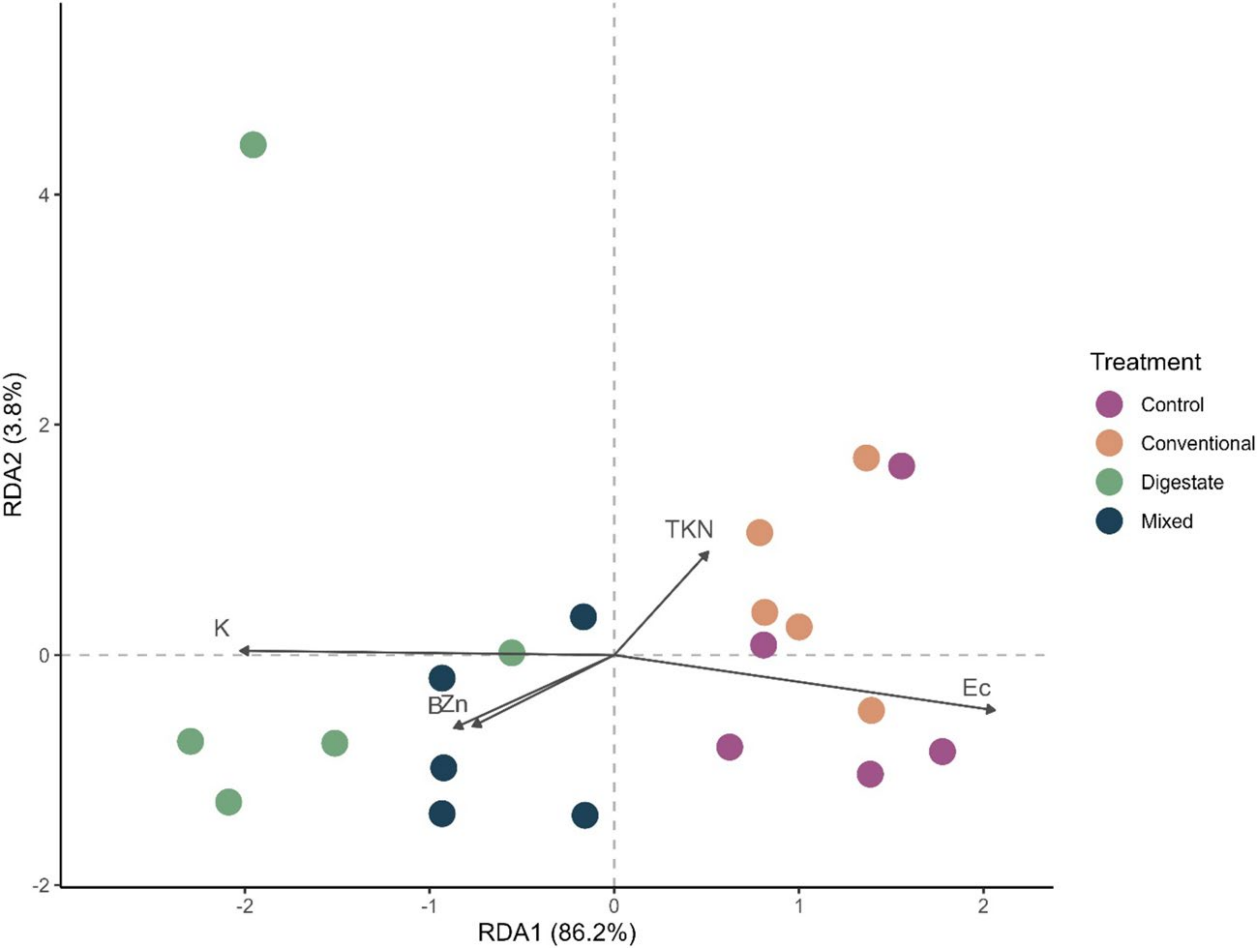
First two axes of redundancy analysis (RDA) using soil chemical properties (predictor variables) and species richness and Shannon diversity indices of nematode and microbial communities (response variable) for different fertilization treatments. Only chemical variables with arrow lengths > 1.9 are showing in the graph. K, potassium; B, boron; Zn, zinc; TKN, total Kjeldahl nitrogen; Ec, electrical conductivity. Different colors represent different fertilization treatments

RDA analysis showed some strong trends between certain chemical properties and fertilization treatments (Fig. 6). The primary axis, explaining the majority of the variability (86%), separates the treatments into two groups: control and conventional treatments on one side of the axis, and digestate and mixed treatments on the opposite side. Most influential factors contributing to this differentiation are TKN and Ec for the conventional and control treatments, respectively, while K, B, and Zn for digestate and mixed treatments. Their significant influence in differentiation can be attributed to their elevated concentration in each treatment, as observed in Fig. 5. This suggests that the higher concentrations of these factors in the respective treatments likely played an important role in shaping the structure of the microbial communities. More specifically, K is a vital nutrient for bacterial survival, maintaining osmotic balance and pH, contributing in protein synthesis, enzyme activation, and electrical signaling (Stautz et al. 2021). Boron facilitates bacterial communication, by synchronizing gene expression, promoting efficient biofilm formation and improving antibiotic production (Breydo 2013). Zinc ions serve as cofactors for bacterial proteins, essential for DNA repair, enzymatic reactions, oxidative stress responses, and regulating various physiological processes (Hantke 2005). Therefore, higher levels of K, B, and Zn could have created an environment favorable to microbial growth and activity and, thus, contributed to the enhanced richness and support of the introduced microbial genera found in digestate-treated soils.

In contrast to the evident impacts on microbial composition, the chemical properties had no substantial effect on nematode populations, either directly or indirectly. Nevertheless, it is possible that the short duration of the experiment might have hindered any significant indirect impacts, such as changes in microbial prey availability, from emerging and altering nematode dynamics.

### Correlation networks

**Fig. 7:**
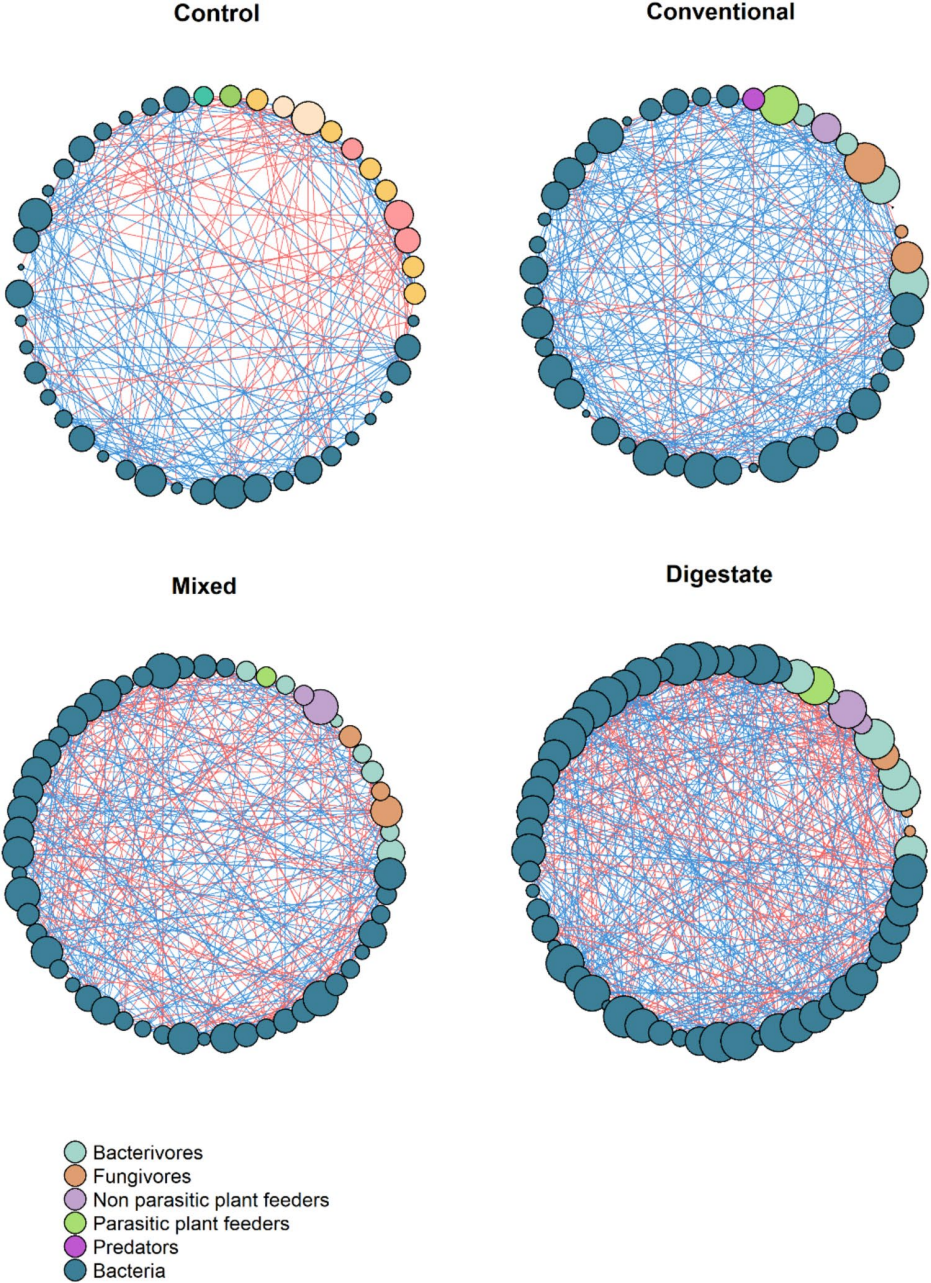
Co-occurrence networks between microbial and nematode genera after different fertilization treatments. Edges represent strong (Spearman’s correlation coefficient > 0.6) correlations. The size of each node is proportional to its degree (the number of connections it has to other nodes). Red and blue edges represent negative and positive co-occurrence relationships

Previous results showed that different fertilization regimes formed different communities. For this reason, a network analysis was performed to investigate the stability and dynamics of each community. Network analysis for the soil communities formed after application of different fertilizers is shown in Fig. 7, while networks’ properties are summarized in Table 2. It is evident that each fertilization regime led the soil community to different pathways and that all networks present small-world properties (SW > 1) and no modularity (M < 0.4). Small-world networks are characterized by high clustering and small path lengths. These properties allow any two members of the microbial community to communicate with each other via a few intermediates.

**Table 2:** The influence of anaerobic digestate and chemical fertilizer on the structural characteristics of interconnected networks involving bacteria and nematodes in the soil. Highest values between treatments are marked with bold

Network properties	Control	Conventional	Digestate	Mixed
Number of edges	244	306	**475**	360
Number of nodes	46	45	**60**	56
Number of positive correlations	160	251	**277**	204
Number of negative correlations	84	55	**198**	156
Positive/negative correlations	1.91	**4.56**	1.41	1.31
Average connectivity (avgK)	10.61	13.6	**15.83**	12.86
Average clustering coefficient (avgCC)	0.54	**0.64**	0.58	0.55
Average path length (APL)	**1.53**	1.49	1.47	1.49
Network diameter	2.89	**3.09**	2.94	2.73
Graph density	0.24	**0.31**	0.27	0.23
Modularity (M)	**0.38**	0.32	0.36	0.35
Cohesion	3	1	6	**7**
Eigenvector centrality	0.52	0.47	0.47	**0.59**
SW	1.95	1.51	1.82	**2.1**
Bacteria-nematodes	88 (36%)	120 (**39%**)	143 (30%)	123 (34%)
Number of positive correlations	21	103	60	77
Number of negative correlations	67	17	83	46
Bacteria-Bacteria	123 (50%)	165 (54%)	314 (**66%**)	221 (61%)
Number of positive correlations	108	133	200	117
Number of negative correlations	15	32	114	104
Nematodes-nematodes	33 (**14%**)	21 (7%)	18 (4%)	16 (5%)
Number of positive correlations	31	15	17	10
Number of negative correlations	2	6	1	6

The control network exhibited fewer nodes and the smallest number of edges, 14% of which are interactions between nematodes. Also, the higher average path length may indicate lower interconnectivity between community members.

Conventional fertilization network presented a rather high positive/negative ratio (4.5), implying a collaborative and mutually beneficial relationship within the community or a lack of antagonism, possibly due to abundance of resources. However, such high ratios are not always beneficial for the overall network stability. It has previously been claimed that the presence of mutualistic associations can disrupt ecosystems by generating co-dependencies (Coyte et al. 2015). This network is also characterized by the largest graph density and average clustering coefficient, reflecting a higher prevalence of tightly interconnected members and therefore quick dispersion of potential perturbations across the network (Zhou et al. 2010).

Digestate-treated network appears to be the densest, with the highest number of edges and nodes. The high degree score of nodes suggests that the nodes within this network are highly interconnected. Interestingly, 66% of total edges in this network result from bacteria-bacteria interactions, underscoring the significance of microbial populations in the formation of the community compared to the other treatments. Furthermore, it exhibits the highest average connectivity, a fact that validates its complexity (Liu et al. 2022).

Mixed fertilization network appears less dense than digestate, with a lower positive/negative ratio (1.31), indicating more balanced facilitative and inhibitive relationships. Additionally, it exhibits the smallest graph density, which is evidence of its reduced complexity, as it has relatively small number of edges compared to the total number of possible edges. The high cohesion of the mixed fertilization network implies a higher degree of connectivity of the community, while the high eigen vector centrality suggests that the community is dominated by many key players.

The higher SW index presented in the mixed network may indicate increased flow of nutrients and energy in the context of resource distribution. Furthermore, because individual nodes can be lost without causing large disturbances to the overall network structure, small-world networks can be more robust and resilient to random and global perturbations. On the other hand, small-world networks are vulnerable to targeted attacks (Papatheodorou et al. 2021). If target central nodes are lost (for example, after the introduction of invasive species), the network will collapse. Based on the aforementioned, it is evidenced that even though the digestate application increased more the genera richness, nevertheless the mixed fertilization regime is the one that presented more stable community structure.

## Conclusions

Application of anaerobic digestate and chemical fertilizer had no major impact on soil microbial structure at the phylum level. Although, the addition of digestate introduced a wide variety of new genera that are known to be involved in nutrient cycling and carbon storage. Surprisingly, the soil microbial diversity remained stable throughout all treatment applications demonstrating a certain resilience of the indigenous microbiota to external disturbances, at least at the doses applied in this study. Chemical fertilization formed the microbial community in a distinct and predictable way in contrast to digestate which had a more stochastic impact. The composition and abundance of nematode community presented no significant differences within the short timeframe of the study. This suggests that the impact of fertilization on nematode populations may require longer-term studies, as many soil nematodes have longer lifespans. The shift of nematode community toward bacterivores in all fertilized soils is probably due to a boost of bacterial abundance, which, in turn, affected the nematode community structure. A tendency of suppression of parasitic plant feeders in digestate-treated soils supports the previously reported nematicidal activity of biogas digestate against this harmful group of nematodes. The most influential chemical properties that drive the community formation were K, B, Ec, and TKN. Digestate and mixed fertilization increased potassium levels in soil, while boron was elevated in the mixed treatment potentially reflecting higher enzyme activities. Surprisingly, both ammonium and nitrate levels were increased in all fertilized soils, regardless of the initial nitrogen units applied, indicating an equivalent nitrogen availability between digestate and chemical fertilizers. Conventional fertilization led to a densely interconnected network with a high positive/negative ratio, potentially indicating mutualistic relationships, but this complexity may lead to ecosystem disruption. However, digestate application led to the formation of a complex community with the highest percentage of bacterial interactions, while the mixed fertilization network showed a more balanced and less complex structure. Overall, the combination of both inorganic fertilizer and digestate reduces the stress levels induced by digestate while simultaneously allowing new taxa with central roles and significant functions in nutrient cycling and stress protection to co-exist with the indigenous community. The soil community formed by this enriched fertilizer is resilient to random disturbances, but on the downside, it is more likely to collapse under targeted perturbations. At this point, it is crucial to emphasize that the observed impacts of fertilizer treatments on microbial and nematode populations are inevitably dependent on the varied dosages applied, which were designed to represent realistic agricultural practices. Thus, application of different dosages may induce divergent responses in the soil biota, as the fertilization dosage is one of the key, most influential parameters that shape directly the soil community and dynamics. Future research should concentrate on longer-term digestate-chemical fertilization treatments to establish if the observed patterns in soil community structure are stable or alter dynamically after repeated applications over time and how a plant-soil system will be affected by the combined fertilization.

## Supplementary Information

Below is the link to the electronic supplementary material.

Supplementary file1 (DOCX 358 kb)

Supplementary file2 (XLSX 17 kb)

## Data Availability

The authors confirm that the data supporting the findings of this study are available upon request from the corresponding author.
