# Supplementary material for: The effect of anaerobic digestate as an organic soil fertilizer on the diversity and structure of the indigenous soil microbial and nematode communities
Source: Environ Sci Pollut Res Int. 2024 Mar 22;32(48):27683–98. doi: 10.1007/s11356-024-32850-9 (PMC12695995; doi:10.1007/s11356-024-32850-9)
Supplement: Supplementary file 1 — Supplementary file1 (DOCX 358 kb) [file 11356_2024_32850_MOESM1_ESM.docx]

**"The effect of anaerobic digestate as an organic soil ~~amendment~~ fertilizer on the diversity and structure of the indigenous soil microbial and nematode communities"**

Charitini Nikolaidou^12^, Magkdi Mola^12^, Spiros Papakostas^3^, Vassilis G. Aschonitis^1^, Nikolaos Monokrousos^2^, Panagiotis G. Kougias^1*^

^1^Soil and Water Resources Institute, Hellenic Agricultural Organization Dimitra, 57001, Thessaloniki, Greece

^2^University Center of International Programmes of Studies, International Hellenic University, 57001, Thessaloniki, Greece

^3^Department of Science and Technology, International Hellenic University, 57001, Thessaloniki, Greece

* Correspondence: Panagiotis G. Kougias, E-mail address: [p.kougias@swri.gr](mailto:p.kougias@swri.gr), Tel: (+30) 2310 473429


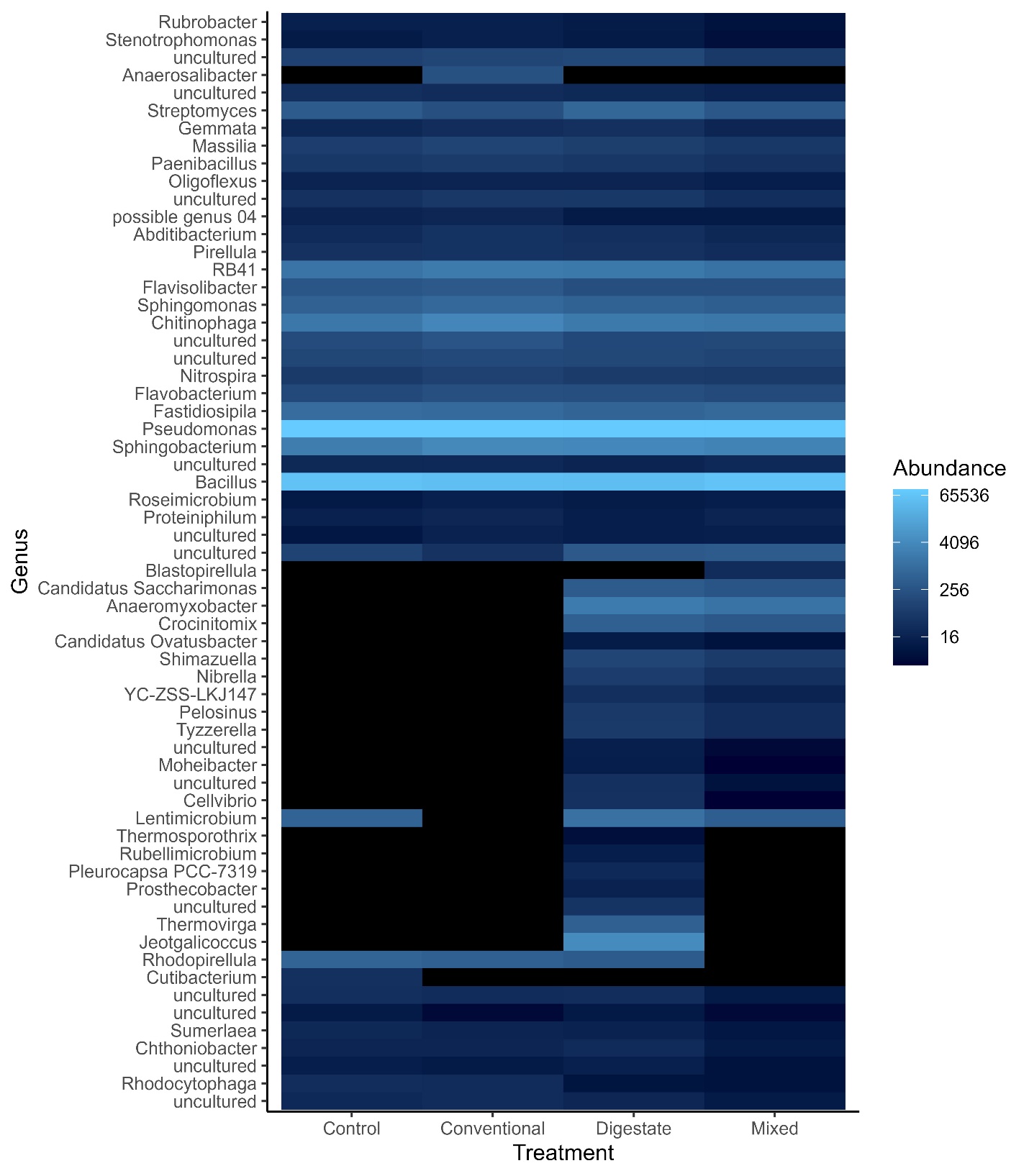


**FigS1** Heatmap depicting the average relative abundance of all taxa in genus level for the treatments tested using Bray-Curtis dissimilarity. Each row in the heatmap represents a specific taxon and each column represents a treatment. Colors represent the scaled relative abundance of taxa with dark blue indicating low abundance and light blue high abundance.
